# Human cytomegalovirus encoded chemokine receptor US28 activates the HIF-1α/PKM2 axis in glioblastoma cells

**DOI:** 10.18632/oncotarget.11817

**Published:** 2016-09-01

**Authors:** Raymond H. de Wit, Azra Mujić-Delić, Jeffrey R. van Senten, Alberto Fraile-Ramos, Marco Siderius, Martine J. Smit

**Affiliations:** ^1^ Division of Medicinal Chemistry, Amsterdam Institute for Molecules Medicines and Systems, Vrije Universiteit Amsterdam, De Boelelaan, Amsterdam, The Netherlands; ^2^ Division of Cell Biology, Faculty of Medicine, Universidad Complutense de Madrid, Madrid, Spain

**Keywords:** G protein-coupled receptor (GPCR), human cytomegalovirus (HCMV), hypoxia-inducible factor (HIF), chemokine, glioblastoma

## Abstract

The human cytomegalovirus (HCMV) encoded chemokine receptor US28 promotes tumorigenesis through activation of various proliferative and angiogenic signaling pathways. Upon infection, US28 displays constitutive activity and signals in a G protein-dependent manner, hijacking the host's cellular machinery. In tumor cells, the hypoxia inducible factor-1α/pyruvate kinase M2 (HIF-1α/PKM2) axis plays an important role by supporting proliferation, angiogenesis and reprogramming of energy metabolism. In this study we show that US28 signaling results in activation of the HIF-1α/PKM2 feedforward loop in fibroblasts and glioblastoma cells. The constitutive activity of US28 increases HIF-1 protein stability through a Gα_q_-, CaMKII- and Akt/mTOR-dependent mechanism. Furthermore, we found that VEGF and lactate secretion are increased and HIF-1 target genes, glucose transporter type 1 (*GLUT1*) and glyceraldehyde-3-phosphate dehydrogenase (*GAPDH*), involved in glucose metabolism, are upregulated in US28 expressing cells. In addition, PKM2 is phosphorylated and found to be in a tumor-associated dimeric state upon US28 expression. Also in HCMV-infected cells HIF-1 activity is enhanced, which in part is US28-dependent. Finally, increased proliferation of cells expressing US28 is abolished upon inhibition of the HIF-1α/PKM2 cascade. These data highlight the importance of HIF-1α and PKM2 in US28-induced proliferation, angiogenesis and metabolic reprogramming.

## INTRODUCTION

The human cytomegalovirus (HCMV) is a widespread pathogen that infects up to 90% of the population [1]. Following a generally asymptomatic primary infection, the virus is able to establish lifelong latencies to evade the host's immune system in healthy individuals. In contrast, during immune-suppression (e.g. AIDS, (auto-) inflammatory disease or allograft patients) the opportunistic virus can cause severe pathology [2-5]. Additionally, HCMV is suggested to act as an oncomodulator during primary infection and/or virus reactivation. A growing body of evidence demonstrates the presence of HCMV in a spectrum of malignant tumors. While HCMV gene products and proteins have been found in colorectal [6, 7], breast [8], prostate [9] and lung tumors [10], among others, the association of HCMV with glioblastoma is most extensively studied [11-16]. It is well established that various human herpesvirus genomes encode G protein-coupled receptors (GPCRs) to support viral pathogenesis [5]. Most of these viral GPCRs show homology to the human chemokine receptor family [17]. Previously, we have shown that the HCMV encoded chemokine receptor US28 promotes tumorigenesis both *in vitro* and *in vivo* by constitutively activating signaling pathways in a G protein-dependent manner, leading to proliferation and angiogenesis [18, 19]. More specifically, US28 constitutively activates NF-κB, increasing COX-2 expression and activity [20]. In addition, US28 is responsible for the secretion of IL-6, thereby activating the transcription factor STAT3 via a positive feedback mechanism involving the cytokine. US28-induced STAT3 activation enhances expression of pro-angiogenic factors such as VEGF [14, 21]. In glioblastoma and medulloblastoma patients, expression of US28 has been exemplified and correlated with increased STAT3/IL-6 as well as COX-2 expression [14, 21, 22].

In order to sustain proliferation and survival of cancer cells, angiogenesis, primarily orchestrated by VEGF, is vital for efficient tumor growth [23]. An important factor known to regulate VEGF expression is the hypoxia-inducible factor 1 (HIF-1). The transcription factor HIF-1 contains an oxygen-regulated α subunit and a stable β subunit, which upon complex formation activates transcription of many genes involved in proliferation (e.g. *TGFA* and *IGF2*), angiogenesis (e.g. *VEGF* and *PDGF*) as well as glycolysis (e.g. *GLUT1*, *HK1* and *PKM2* [24]. In cancer cells, expression of oxygen-regulated subunit (HIF-1α) is increased by either increased HIF-1α protein synthesis and stability or by increased mRNA levels [24]. Reprogramming of energy metabolism is a hallmark of cancer, transformed cells switch from the slow yet energetically favorable oxidative phosphorylation towards the fast and less glucose-efficient aerobic glycolysis to generate ATP [25]. Pyruvate kinase M2 (PKM2) is an important enzyme in energy metabolism, since it converts phosphoenol pyruvate (PEP) and ADP to pyruvate and ATP [26].The glycolytic enzyme *PKM2* is both a HIF-1 target gene and a regulatory protein of HIF-1 activity. The protein kinase activity and co-transcription factor function of *PKM2* stimulate HIF-1α activity and expression, respectively. Thus, HIF-1α and PKM2 engage in a feedforward loop, enhancing activity of both key metabolic regulatory proteins [27-29].

In this study we demonstrate that the HCMV-encoded chemokine receptor US28 stimulates the HIF-1/PKM2 feedforward loop, resulting in increased cell proliferation, VEGF secretion and glycolysis in fibroblasts and glioblastoma cells. Also in HCMV-infected cells, US28 mediates increased HIF-1α activity. These observations further confirm the oncomodulatory role of US28, which through HIF-1α and PKM2 drives cell proliferation, angiogenic processes and metabolic reprogramming.

## RESULTS

### US28 mediates increased VEGF secretion involves HIF-1 activation in (pre-) malignant cells

**Figure 1:**
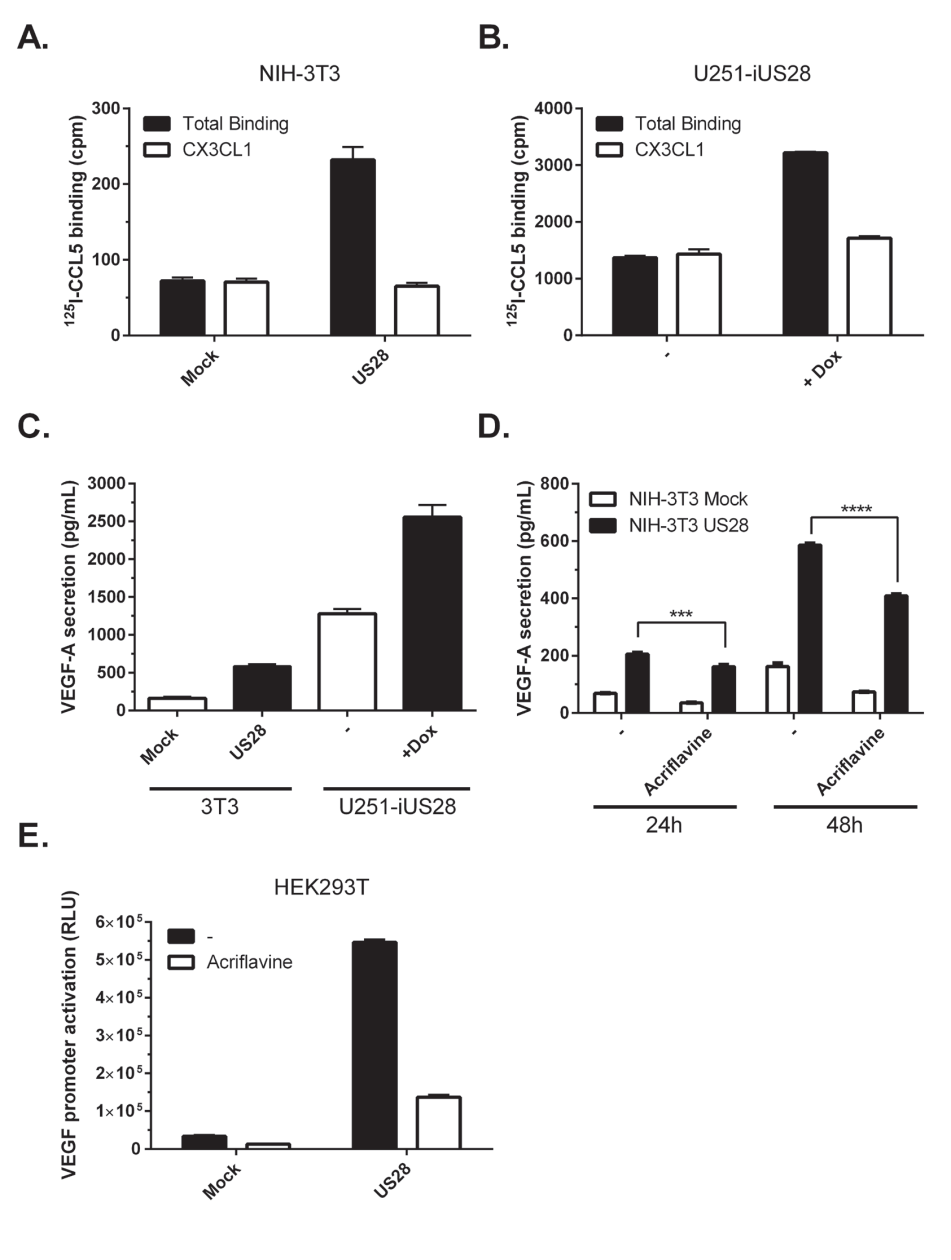
US28 constitutively induces promoter activation and secretion of VEGF via HIF-1 NIH-3T3 cells stably expressing US28 **A.** or U251 cells with inducible expression of US28 **B.** were assayed for binding of radiolabeled ^125^I-CCL5 (200 pM) and displacement with unlabeled US28 ligand CX3CL1 (100 nM). **C.** 48 hour secretion of VEGF-A in the supernatant of synchronized mock and US28-expressing NIH-3T3 or U251 cells. **D.** VEGF-A secretion after 24/48 hour incubation with or without HIF-1 dimerization inhibitor acriflavine (1 μM) treatment. Significant differences between conditions are depicted by asterisks (*** = *P* < 0.001, **** = *P* < 0.0001). **E.** HEK293T cells transfected with US28 and VEGF promoter reporter gene were treated with HIF-1 dimerization inhibitor acriflavine (1 μM), VEGF promoter activity was measured after 24 hours.

Previously, we demonstrated that the HCMV-encoded receptor US28 constitutively promotes tumorigenesis in NIH-3T3 cells, among other mechanisms, through secretion of VEGF [18]. In tumor tissue samples from glioblastoma patients we also detected US28 expression, indicating a potential role for the viral GPCR in HCMV-infected tumors [21]. To study the role of US28 in more detail we evaluated its effects in pre-malignant fibroblasts (NIH-3T3) and disease-relevant malignant glioma cells (U251), to define the role of the receptor in different stages of cancer development. To this end an inducible U251 glioblastoma cell line was generated with Tet-repressor regulated US28 expression (U251-iUS28). Constitutive or doxycycline-induced US28 expression was detected by specific ^125^I-CCL5 displacement, a chemokine known to bind US28, on NIH-3T3 and U251-iUS28 cells, respectively (Figure 1A, 1B). US28 expression resulted in elevated secretion of VEGF in both cell lines (Figure 1C). The basal levels of VEGF secretion were, as expected, much higher in U251 glioma cells compared with the non-malignant NIH-3T3 fibroblasts. US28 expression in U251 cells resulted in a pronounced increase of VEGF secretion, albeit with a relative smaller fold-increase compared to NIH-3T3 cells (2.2 ± 0.1 *vs*. 3.3 ± 0.2). Since HIF-1 is known to regulate VEGF expression we investigated the role of HIF-1 in US28-induced VEGF secretion. Acriflavine, an inhibitor of HIF-1 dimerization [30], significantly inhibited VEGF secretion after 24 and 48 hours of treatment (Figure 1D). In HEK293T cells transiently expressing US28, VEGF promoter activation was increased, which was partially inhibited by acriflavine treatment (Figure 1E). These findings indicate the involvement of HIF-1 in US28-induced VEGF expression.

### HIF-1α protein levels are increased under normoxic conditions in US28-expressing cells

**Figure 2:**
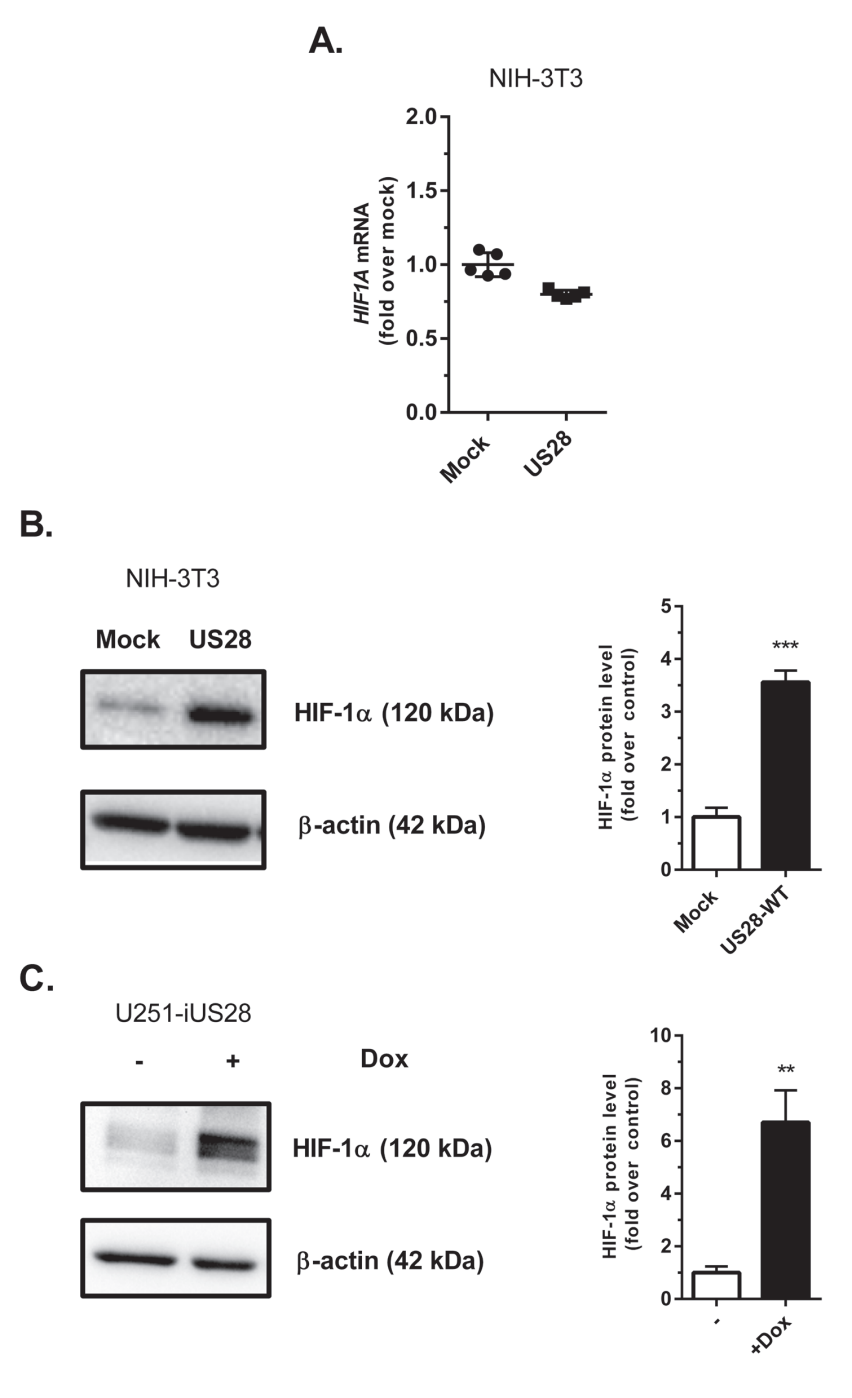
US28 constitutively induces the transcriptional activity of the HIF-1 complex by increasing the HIF-1α protein level **A.** Stably transfected mock and US28 NIH-3T3 cells were used for RNA isolation. Quantitative RT-PCR is performed to determine *HIF1A* relative mRNA expression levels, using *ACTB* as normalization control. Lysates were prepared from stably transfected NIH-3T3 cells with US28 or control vector **B.** or U251-iUS28 cells with inducible US28 expression **C.**, expression of HIF-1α protein was evaluated with a specific antibody by Western blotting, followed by signal quantification using β-actin expression as a loading control. Significant differences between conditions are depicted by asterisks (** = *P* < 0.01, *** = *P* < 0.001)

Under normoxic conditions HIF-1α is usually rapidly degraded in healthy cells by an efficient oxygen-regulated mechanism [31]. In cancer cells, however, HIF-1α protein levels are generally increased, independent of the oxygen level, thus stimulating HIF-1α/HIF-1β transactivation and regulation of HIF-1 target genes. Since HIF-1β is constitutively expressed, upregulation of HIF-1α level is the determining factor enhancing HIF-1 complex transactivation. The effects of US28 expression on HIF-1α gene transcription and protein level were evaluated under normoxic conditions (Figure 2). As depicted in Figure 2A, NIH-3T3 cells showed no relevant change in HIF-1α transcription, represented by mRNA transcript levels of mock and US28-expressing cells. However on the protein level, a clear upregulation of HIF-1α was observed in NIH-3T3 cells constitutively expressing US28 (Figure 2B). A 48 hours induction of US28 expression in synchronized U251 glioma cells similarly resulted in HIF-1α upregulation (6.7 ± 1.2 fold increase) (Figure 2C). Since HIF-1α undergoes post-translational modifications that affect activity and stability [31], HIF-1α is detected by multiple protein bands in the 100-120 kDa range. Overall the data demonstrates that US28 expression in fibroblasts and malignant glioma cells results in an extensive increase of HIF-1α protein levels, whereas gene transcription is unaffected. This indicates a direct mechanistic link between US28 signaling and HIF-1 activation potentially in different stages of tumor development.

### US28-dependent Gαq protein signaling increases HIF-1 transactivation through activation of CaM-KII and Akt/mTOR pathways

**Figure 3:**
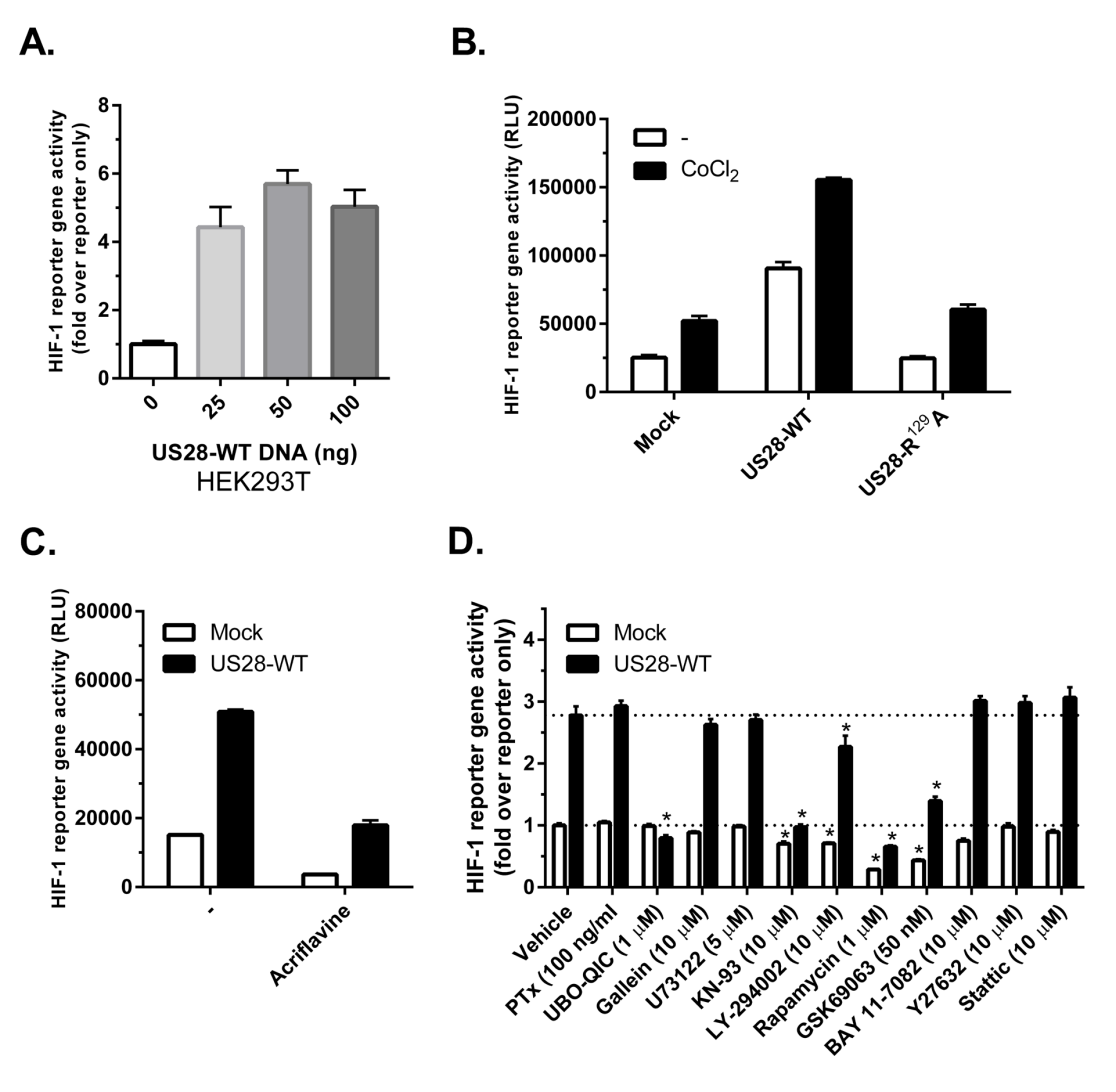
US28 stimulates HIF-1 transcriptional activity through concerted activation of Gα^q^, CaMKII and the Akt/mTOR signaling cascade **A.**-**D.** HEK293T cells were transiently co-transfected with US28-encoding DNA and 500 ng of hypoxia response element (HRE) HIF-1 reporter gene, transcriptional activity was measured 24 hours after transfection and treatment. **A.** Increasing amounts of US28-WT DNA were transfected with a fixed amount of reporter gene **B.** Transfection of US28-WT or the G protein-uncoupled mutant US28 R^129^A and treatment with cobalt chloride (150 μM) to mimic hypoxia **C.** Treatment with HIF-1 dimerization inhibitor acriflavine (1 μM). **D.** A panel of pharmacological inhibitors is applied to inhibit US28-dependent HIF-1 activation in a HIF-1 reporter gene assay using HEK293T cells. US28-expressing and mock (reporter only) cells were treated with inhibitors for 24 hours before activity was measured. The results normalized to the vehicle condition of the reporter only control. The dotted lines represent the basal level of US28-expressing or mock cell reportergene activity. Significant (α=0.05, *P* < 0.05) reporter gene activity inhibition compared to the vehicle controls is represented by an asterisk (*) above the bar.

In order to elucidate the mechanism by which US28 controls HIF-1, a hypoxia response element (HRE) luciferase reporter gene was used. Because the HIF-1 complex associates with HRE sequences of target gene promoter regions, the reporter gene luminescence accurately reflects HIF-1 transcriptional activity. Transient co-transfections of HEK293T cells with increasing amounts of US28 DNA and a fixed amount of the HRE reporter gene revealed a dose-dependent increase in luminescence, indicating that US28 is able to constitutively stimulate HIF-1 activity (Figure 3A). In contrast, HIF-1 activation was not observed in cells expressing the G protein-uncoupled receptor mutant US28-R^129^A (US28-R^3.50^A, according to Ballesteros-Weinstein nomenclature), demonstrating that US28-induced HIF-1 activity is G protein-dependent. HIF-1 reporter gene activity could be further induced in mock and US28 expressing cells by chemically mimicking hypoxia using CoCl_2_ [32] (Figure 3B). A direct inhibition of HIF-1 transactivation resulted in an apparent decrease of basal and US28-dependent reporter gene activity (Figure 3C). Taken together, these data show the involvement of US28 signaling in stimulation of HIF-1 complex activity to regulate expression of HRE target genes. Next, the mechanism of US28-dependent HIF-1 activation was addressed, which potentially involves a previously undescribed signaling cascade. US28 is known for its pleiotropic activation of proliferative and inflammatory signal transduction pathways, including activation of many transcription factors, such as NF-κB, NFAT, TCF-LEF and STAT3 [5]. HIF-1 reporter gene assays were conducted in the presence of pharmacological inhibitors targeting key proteins of US28 and/or HIF-1 signaling. Since US28 promiscuously interacts with several G protein families [5], the G protein family-specific inhibitors pertussis toxin (Gα_i/0_) and UBO-QIC (Gα_q/11_), and the βγ-subunit inhibitor gallein were included in the reporter gene assay. Using pathway inhibitors the involvement of PI3K/Akt/mTOR (inhibitors; LY-294002, GSK690693, rapamycin) PLC-β/PKC (inhibitors; U73122, Bisindolylmaleimide I, KN-93), RhoA/ROCK (inhibitors; Y-27632, H89), JAK/STAT3 (inhibitors; P6, Stattic) and NF-κB (inhibitor; BAY11-7082) signaling was determined. Figure 3D depicts the HIF-1 reporter gene activities of US28- and mock-transfected (i.e. reporter gene only) HEK293T cells with a number of inhibitors, relative to the vehicle-treated mock control. An overview of the applied inhibitors is portrayed in Table I, showing the pharmacological target, percentage of HIF-reportergene inhibition with mock and US28 transfected cells. Multiple t-tests were performed to indicate whether there is a significant difference between the percentage inhibition by the inhibitors in mock and US28-expressing cells associated with US28-selective inhibition (Table I). The US28 induced HIF-1 activity can be fully inhibited to mock levels with the Gα_q/11_-specfic inhibitor UBO-QIC. Pertussis toxin or gallein treatment, in contrast, do not affect US28-dependent HIF-1 activity (Figure 3D). Together this illustrates that US28-dependent HIF activity is mediated through Gα_q/11_ signaling. Interestingly, CaMKII activation appears to be paramount in US28-dependent HIF-1 signaling, since KN-93 treatment results in effective (65%) and specific inhibition of US28-dependent HIF-1 activation (Figure 3D). Downstream of CaMKII, US28 signaling appears to feed into the canonical Akt/mTOR cascade of HIF-1 activation, since GSK690693 (Akt) and rapamycin (mTOR) strongly impair US28-dependent and basal HIF-1 reporter gene activity (Figure 3D, Table I). LY-294002 treatment results in a weak and non-selective, yet significant signal inhibition (Figure 3D, Table I). The NF-κB inhibitor (BAY 11-7082) and JAK/STAT3 pathway inhibitors (P6, Stattic), previously shown to inhibit US28 mediated STAT3 activity [20, 21], do not impair US28-dependent HIF-1 activation (data not shown).

The pharmacological inhibitors that inhibit HIF-1 reportergene activity were also tested in an upstream inositol-phosphates (IP) accumulation assay (data not shown). The experiments revealed that only the Gα_q_ inhibitor (UBO-QIC) attenuated US28-dependent IP-accumulation, the other pharmacological inhibitors had no effect in this assay. This shows that the pharmacological inhibitors do not induce acute cell toxicity. In order to confirm the abovementioned findings, we next investigated the effects of the pharmacological inhibitors in more disease-relevant models and assay read-outs, which will be discussed in detail below.

**Table 1:** Overview of US28 HIF-1 reportergene data.

Inhibitor	Target	Reporter only inhibition (%)	US28 inhibition (%)	US28-selective inhibition (p value)
Pertussis Toxin	Gα_i_	−5	−5	0,886
UBO-QIC	Gα_q_	1	71	1,30E-06
Gallein	Gβγ	11	2	0,057
U73122	Phospholipase C	2	2	0,928
KN-93	CaMKII	30	65	1,20E-04
LY-294002	PI3K	29	18	0,145
Rapamycin	mTOR	71	76	0,024
GSK690693	Akt	56	50	0,084
BAY 11-7082	NF-κB	9	−1	0,581
H89	PKA/ROCK	30	43	0,027
Y27632	ROCK	2	−7	0,224
P6	JAK	0	−3	0,640
Stattic	STAT3	11	−11	0,021
Bisindolylmaleimide	PKC	68	68	0,862

The applied inhibitors and their pharmacological targets, inhibition of transcriptional activity in HIF-1 reporter only transfected cells and inhibition on US28 and HIF-1 reporter transfected cells. Multiple t-tests with Holm-Sidak correction were performed to determine statistically significant (α=0.05) US28-selective inhibition (*P* value).

### US28 promotes transcription of HIF-1 target genes and reprograms Akt and PKM2 activity in fibroblasts and glioma cells

**Figure 4:**
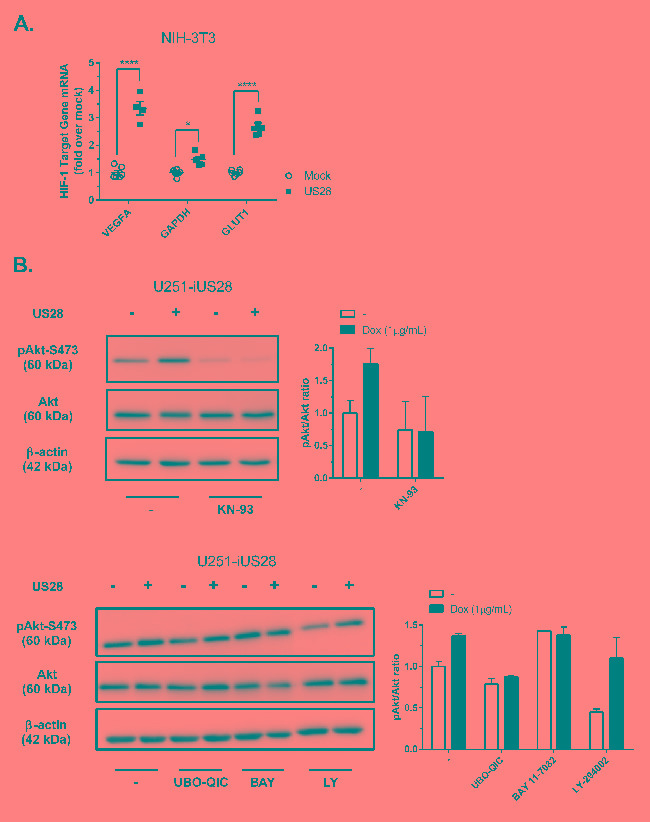

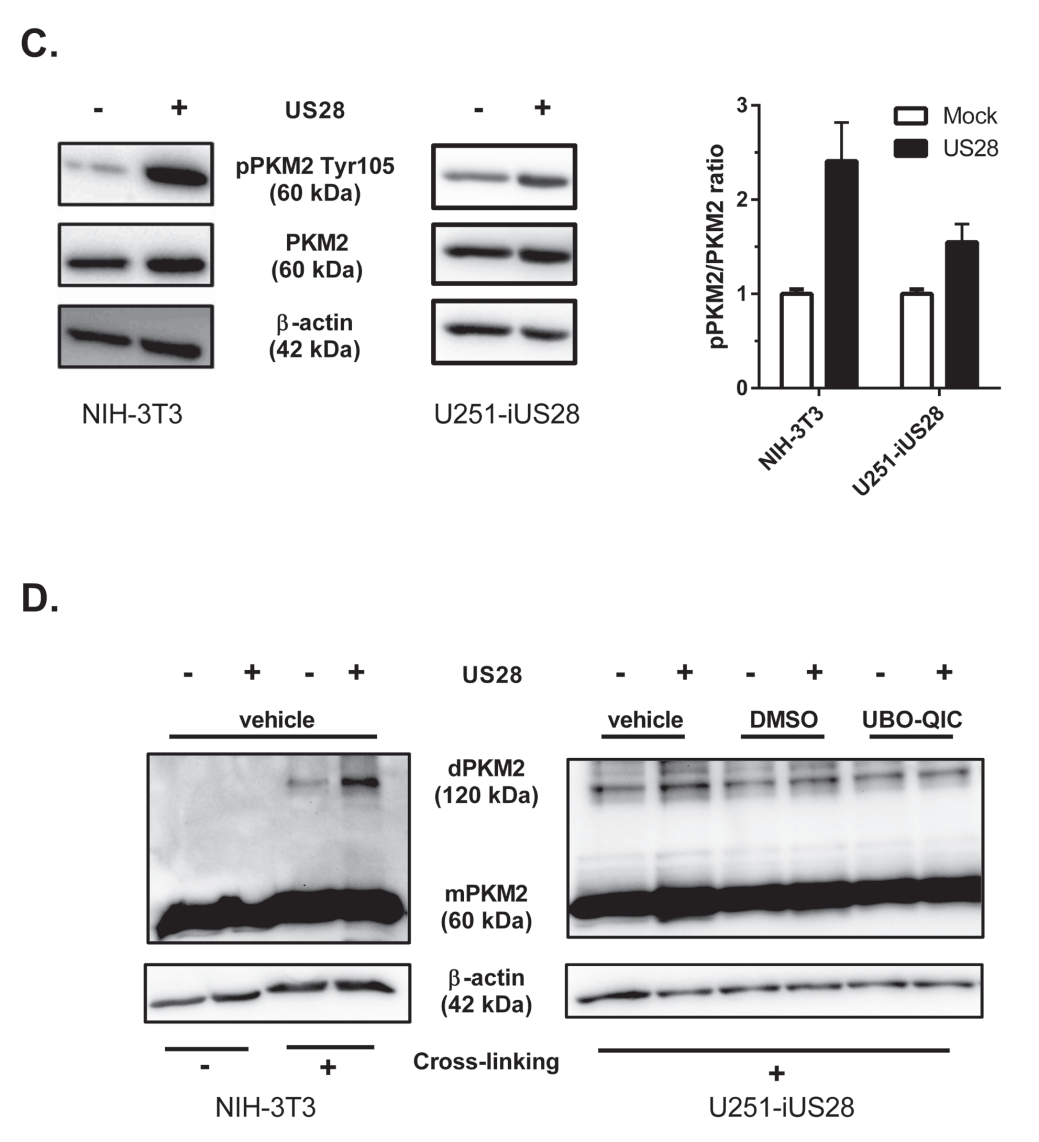
US28 promotes HIF-1 target gene transcription and reprograms activation of Akt and PKM2 which are involved in proliferation, angiogenesis and glucose metabolism through a Gα_q_/CaMKII/Akt/HIF-1 dependent fashion in fibroblasts and glioma cells **A.** HIF-1 target genes *GAPDH*, *GLUT1* and *VEGFA* relative mRNA levels were determined by quantitative RT-PCR using RNA isolated from US28 or mock transfected NIH-3T3 cells and gene-specific primers. Significant differences in HIF-1 target gene expression are depicted by asterisks (* = *P* < 0.05, **** = *P* < 0.0001). **B.** Lysates were prepared from synchronized U251-iUS28 cells with/without 48 hours of doxycycline-induced US28 expression and treatment with signaling pathway inhibitors. SDS/PAGE Western blot analysis for phosphorylated Akt (Ser 473), total Akt and the loading control β-actin. Relative Akt activity is semi-quantified by pAkt/Akt ratios **C.** Lysates were prepared from synchronized, stably transfected NIH-3T3 or U251-iUS28 cells and phosphorylation status of PKM2 were assessed by Western blotting. Relative PKM2 phosphorylation is semi-quantified by pPKM2/PKM2 ratios **D.** Native lysates were prepared from synchronized US28 or mock NIH-3T3 and U251-iUS28 cells. For U251-iUS28, native lysates were prepared after 48 hours of US28 expression with/without vehicle, DMSO or UBO-QIC treatment. PKM2 protein was cross-linked using 0.25% formaldehyde/PBS solution (+), lysates without cross-linking treatment (−) were included as loading and total PKM2 expression controls. PKM2 and β-actin expression levels were assessed by Western blotting.

Solid tumors are generally characterized by increased glucose uptake and metabolism [33], HIF-1 transcriptional activity contributes to this phenotype by regulating the gene transcription of, amongst others, *glyceraldehyde-3-phosphate dehydrogenase* (*GAPDPH)*, *glucose transporter type 1* (*GLUT1)*, *lactate dehydrogenase A* (*LDHA)* and *pyruvate kinase M2* (*PKM2)*, all involved in glucose uptake and metabolism [34-36]. Since increased HIF-1α protein levels (Figure 2) and transcriptional activity (Figure 3) were observed, we next assessed mRNA relative expression levels of HIF-1 target genes *VEGFA*, *GAPDH* and *GLUT1* in NIH-3T3 cells by quantitative RT-PCR. As expected from the measurements of VEGF promotor activity and secretion (Figure 1), the level of mRNA encoding the angiogenesis regulator *VEGFA* was increased in US28-expressing NIH-3T3 cells (Figure 4A). Additionally, *GAPDH* and *GLUT1* mRNA expression were also significantly upregulated for these glucose metabolism genes in cells expressing US28 (Figure 4A). These findings suggest that US28 may drive reprogramming of glucose metabolism in pre-malignant cells.

The reporter gene data obtained from US28 expressing HEK293T cells, suggested the involvement of the Akt/mTOR signaling cascade in regulating US28-dependent HIF-1 activity in these cells (Figure 3E). Since Akt is reported to play a role in HIF-1 translation and transcription [37], we studied the role of active Akt (phosphorylated at Ser473) in US28-expressing cells. The phosphorylated Akt/total Akt (pAkt/Akt) ratios were determined to assess the relative level of Akt activity and signaling. After 48 hours of doxycycline-induced US28 expression in U251 cells, an increase of Akt phosphorylation at Ser473 (ratio: 1.8 ± 0.2) was detected, which could be inhibited by Gα_q_ (UBO-QIC) and CaMKII (KN-93) inhibitors (Figure 4B). NF-κB (BAY 11-7082) or PI3K (LY-294002) inhibition did not attenuate US28-dependent Akt phosphorylation in U251 cells, which is in line with the HIF-1 reportergene data. BAY 11-7082 (NF-κB inhibitor) treatment resulted in an increase of Akt activity, regardless of US28 expression. This indicates a non-specific and/or US28 unrelated effect of this inhibitor on basal Akt activity. Since BAY 11-7082 treatment on US28-expressing cells did not suppress relative Akt phosphorylation this implies that US28 stimulates Akt signaling independently of NF-κB. PI3K inhibition resulted in a decrease of basal Akt phosphorylation, however, US28-dependent Akt phosphorylation was unaffected. These data illustrate that US28 signaling promotes non-canonical (PI3K-independent) Akt activation.

The combined observations of increased HIF-1 protein levels and transcriptional activity, together with increased Akt/mTOR activity, led to the hypothesis that US28 might enhance glycolysis. To test this hypothesis, we examined the phosphorylation and oligomeric status of PKM2, a crucial regulatory enzyme in the glycolytic pathway. PKM2 functions as a metabolic master switch with a distinct dual role depending on its oligomeric form. Tetrameric PKM2 functions as a pyruvate kinase in the cytosol, driving the energetically favorable cycle of oxidative phosphorylation [38]. PKM2 phosphorylation at Tyr105 was shown to inhibit formation of the active tetramer and promotes dimerization of the protein [39]. The PKM2 dimer displays reduced pyruvate kinase activity and is associated with increased glycolytic flux and lactate secretion [39]. In addition, dimeric PKM2 is able to translocate to the nucleus where it functions as a protein kinase and/or co-transcription factor, regulating expression of proliferative genes thereby promoting tumorigenesis [28, 38, 40]. In US28-expressing NIH-3T3 cells there is an evident (3-fold) increase in Tyr105 phosphorylation of PKM2, with only a modest (roughly 10%) increase in PKM2 protein levels (Figure 4C). This suggested a switch of PKM2-dependent pyruvate kinase activity towards an increase in protein kinase and co-transcriptional activity in these US28 expressing NIH-3T3 cells, compared to control cells. A similar trend was observed in U251 cells with inducible US28 expression, albeit to a lesser extent compared to NIH-3T3 cells. US28 expression results in a marked increase of phosphorylated PKM2 in the U251 glioma cells (1.55 ± 0.19) (Figure 4C). PKM2 cross-linking experiments were performed to elucidate if the abundance of PKM2 dimers is altered by US28 expression. Native lysates of stably transfected NIH-3T3 cells and U251 cells with inducible US28 expression were subjected to formaldehyde cross-linking and subsequently probed for PKM2 levels by Western blot analyses (Figure 4E). Without cross-linking the PKM2 antibody shows a specific interaction at 60 kDa, the size of monomeric PKM2. Upon crosslinking the proteins in the cell lysates, an additional signal appears at 120 kDa, the expected molecular weight of dimeric PKM2. The crosslinking conditions with a low formaldehyde concentration (0.25%) were highly stringent, since no additional PKM2 signals could be detected between 60 and 120 kDa in molecular size. There is an increase of dimeric (120 kDa) PKM2 in US28-expressing cells compared to mock cells in NIH-3T3 and U251 cell lines. In doxycycline-treated U251-iUS28 cells, inhibition of Gα_q_ by UBO-QIC resulted in decreased dimeric PKM2 protein levels, again illustrating the involvement of this G-protein in US28-dependent PKM2 regulation, in line with the previous HIF-1 reportergene and Akt phosphorylation data. All together the data supports the hypothesis that US28 stimulates the metabolic master switch PKM2 to be in a dimeric conformation enhancing glycolysis and promoting cell proliferation.

### US28 stimulates cell proliferation and initiates reprogramming of glucose metabolism

**Figure 5:**
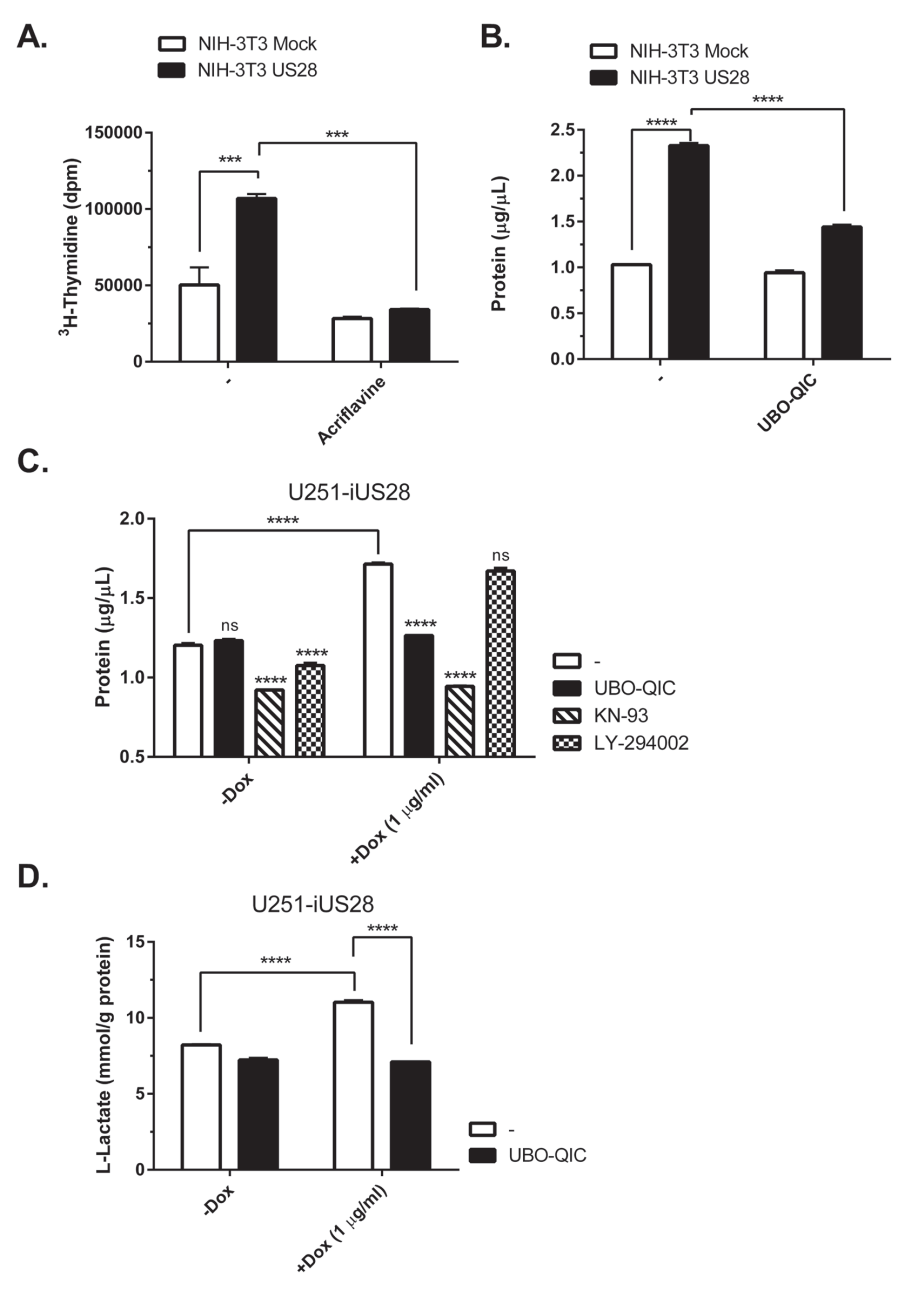
US28 signaling results in increased cell proliferation and metabolic reprogramming **A.** Stably transfected NIH-3T3 cells with US28 or empty vector were treated with acriflavine for 48 hours. Proliferation of the cells was determined by 3H- thymidine incorporation assay. **B.** Mock and US28 NIH-3T3 cells were synchronized and subsequently treated with UBO-QIC for 48 hours before lysates were prepared and protein content was determined using a BCA assay **C., D.** Synchronized U251 cells with inducible US28 expression were cultured for 48 hours with/without doxycycline and pharmacological inhibitors. (D) The lysate protein content was determined using a BCA assay, **E.** whereas cell culture medium supernatant was analyzed for L-Lactate levels using a cell-based glycolysis assay. The L-Lactate levels are normalized to the total protein content. Significant differences between conditions are depicted by asterisks (***= *P* < 0.001, **** = *P* < 0.0001).

Previously, we have shown that NIH-3T3 cells stably expressing US28 display increased cell growth and proliferation [18]. To determine whether activation of the HIF-1/PKM2-axis is of importance in US28-dependent cell growth, ^3^H-thymidine incorporation was measured after treatment with the HIF-1 dimerization inhibitor acriflavine (Figure 5A). Acriflavine treatment resulted in decreased proliferation of cells expressing US28, compared to untreated cells. As cell proliferation correlates to increased biomass, we next assessed the US28-dependent differences in total protein content of NIH-3T3 and U251 cell lysates. US28-expressing cells demonstrated a greater increase in biomass in both cell lines (Figure 5B, 5C). In NIH-3T3 cells, Gα_q_ inhibition with UBO-QIC resulted in selective inhibition of biomass increase in US28-expresssing cells (Figure 5B). US28-dependent U251 cell proliferation could be inhibited by the pharmacological inhibitors UBO-QIC (Gα_q_) and KN-93 (CaMKII), whereas there was no significant effect of LY-294002 (PI3K) treatment in these cells (Figure 5C). This highlights that US28 drives Gα_q_/CaMKII/Akt/HIF-1 signaling to support cell proliferation. Increased cell proliferation of malignant cells frequently relies on increased glycolysis to generate ATP, which is classically known as the Warburg effect [41]. Glycolysis, a rapid yet energetically inefficient mechanism of glucose metabolism is concomitant with increased lactate production and secretion. Also in glioblastoma, glycolysis plays a prominent role and intervention in the glycolytic cascade is considered as a viable therapeutic approach targeting this disease [42]. Since US28 upregulates glycolytic HIF-1 target genes and modulates of PKM2 functionality (Figure 4), we postulated that US28 signaling affects glycolysis and thus lactate production in U251 glioma cells. This was confirmed by measuring increased L-lactate levels in conditioned medium of iUS28-U373 cells 48 hours after stimulation of expression of this viral GPCR. The elevated level of lactate secretion in US28-expressing cells was reduced to control levels by Gα_q_ inhibition (Figure 5D). Taken together, these data indicate that US28 signaling results in increased HIF-1-dependent cell proliferation of NIH-3T3 and U251 cells, which is accompanied by reprogramming of glucose metabolism indicated by an elevation of glycolysis.

### HCMV infection induces HIF-1 activity in part via US28

**Figure 6:**
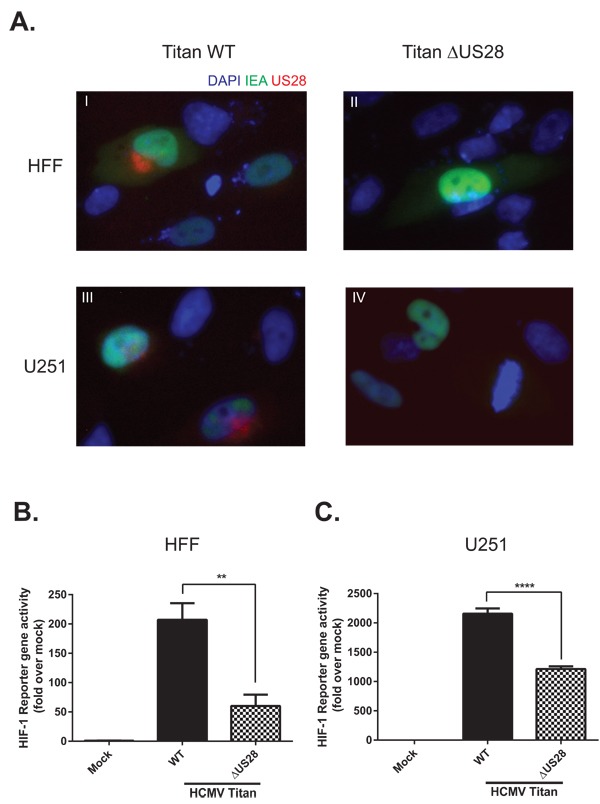
US28 contributes to HCMV-dependent HIF-1 activation in U251 glioblastoma and HFF cells **A.** Immunofluorescence of HCMV Titan WT and ΔUS28 infected U251 or HFF cells 48 hours post-infection at MOI2. Cells were stained with US28 antibody (red fluorescence), anti-immediate early antigen (IEA) antibody (green fluorescence) and DAPI (blue fluorescence). Additionally, since a GFP tag is incorporated in the viral genome, the cytosol of infected cells can also be detected with green fluorescence. Panel I-IV display IEA-specific staining in the nuclei and GFP in the cytosol of HCMV Titan-infected cells. Panels I and II display US28-specific staining in primarily the perinuclear region of Titan WT infected cells, which cannot be detected in cells infected with the US28 deletion mutant (ΔUS28). **B.** HFF or **C.** U251 cells were infected with HCMV Titan WT or ΔUS28 at MOI of 2. Mock and infected cells were transfected with the HRE reporter gene 24 hours post-infection, followed by HIF-1 activity measurement 48 hours post-infection. Significant differences between conditions are depicted by asterisks (** = *P* < 0.01, **** = *P* < 0.0001).

As US28 is an HCMV-encoded GPCR it is essential to study its signaling properties in a viral context. McFarlane and colleagues described the ability of HCMV to induce HIF-1 [43], yet the viral proteins and molecular mechanism driving this process remain unclear. To improve our understanding of HCMV-dependent HIF-1 induction, we studied the contribution of US28 to HIF-1 activity upon HCMV infection of relevant cell lines. Human foreskin fibroblasts (HFF) and U251 malignant glioblastoma cells were infected with the clinical HCMV-Titan strain (Titan WT) and its US28 deletion mutant (Titan ΔUS28) [18] at a multiplicity of infection (MOI) of 2 and assessed for viral protein (Immediate early antigen (IEA) and US28) expression and HIF-1 activity 48 hours post-infection (Figure 6). Immunofluorescence of HCMV Titan WT and ΔUS28-infected HFF and U251 were performed to verify infection efficiency and US28 expression (Figure 6A). The infected cells can be visualized by IEA staining in the nucleus (green fluorescence). The genomes of HCMV Titan strains encode GFP, resulting in minor cytosolic expression levels of this fluorescent protein (also green fluorescence) during later phases of the infection cycle. The representative immunofluorescence images (panel I-IV) illustrate that HFF and U251 cells are susceptible to HCMV Titan WT and ΔUS28 infection. As described previously, US28 (red fluorescence) is only expressed in the WT-infected cells (panel I and III) and primarily localized to the perinuclear region [21, 44]. As can be seen in Figures 6B and 6C, the HRE reporter gene results of infected HFF (Figure 6B) and U251 (Figure 6C) cells indicate that HCMV Titan WT infection induces HIF-1 transcriptional activity to a great extent, which is partially, but significantly reduced in cells infected with the Titan ΔUS28 strain. Thus, demonstrating that HCMV-dependent HIF-1 transactivation is in part attributed to US28 signaling. Co-transfection of HFF and U251 cells with a constitutive *Renilla* luciferase reporter as control showed similar *Renilla* luciferase activities in all conditions (data not shown), demonstrating that HCMV infection and US28 expression do not affect the basal transcription machinery. Altogether, the HCMV infection experiments demonstrate that US28 affects HIF-1 transactivation in a viral context.

## DISCUSSION

HIF-1α is an oxygen-sensitive regulator of many key cellular processes such as cell proliferation and survival, metabolism and angiogenesis. In normoxia, HIF-1α protein levels are low due to concerted action of hydroxylases, which promote interaction with ubiquitin ligases, marking the transcription factor for proteosomal degradation [31]. Under hypoxic conditions, oxygen-dependent hydroxylases fail to promote HIF-1α degradation, resulting in its translocation to the nucleus where it forms a functional dimer with HIF-1β and regulates transcription of over 100 HRE genes [45]. Increase of HIF-1α protein levels allows for the activation of many genes that will provide the required building blocks for rapid cell growth [46]. Cancer cells are able to increase HIF-1α levels even under normoxic conditions. This occurs mostly through mutations of regulatory proteins resulting in activation of receptor tyrosine kinases and/or GPCRs or loss of tumor suppressor function (e.g. p53 and PTEN). Besides mutations, infection with oncogenic viruses has been reported to alter HIF-1α regulation, thereby contributing to the oncogenic phenotype. KSHV is known to increase HIF-1-mediated transactivation as well as nuclear localization and decrease protein degradation [47-50]. This is, partially, attributed to the KSHV-encoded GPCR ORF74 that induces HIF-1 transactivation under non-hypoxic conditions [46, 49]. Other examples of oncogenic viruses increasing HIF-1 activity are Hepatitis B and C virus [51, 52], EBV [53, 54], HPV [55] and HCMV [43].

Although the precise role of HCMV in tumor etiology and development remains elusive, the virus and more specifically its encoded viral GPCR US28 have been ascribed an oncomodulatory role in tumorigenesis [5, 12, 14]. In this study we evaluated the role of US28 in the context of glioblastoma, a highly malignant tumor which is frequently positive for HCMV [56]. We demonstrate that HCMV-encoded viral GPCR US28 mediates proliferation, angiogenic processes and metabolic reprogramming via activation of HIF-1α. Inhibition of HIF-1α results in impaired proliferation and VEGF secretion in US28-expressing cells (Figure 1 and 5, respectively). In line with earlier observations [43], HCMV-infected human foreskin fibroblasts and glioblastoma cells displayed increased HIF-1 transcriptional activity that could be attributed to US28 (Figure 6). Moreover, McFarlane *et al.* showed that inactivated HCMV, irradiated with ultraviolet light, also induced HIF-1, demonstrating that the response was elicited by interaction of the infecting virion with the cell and that *de novo* viral gene expression was not required. A viral factor involved could be US28, since it is expressed on the virion [57]. In cells infected with the HCMV Titan ΔUS28 strain, significant residual HIF-1 activity remained, suggesting other HCMV-encoded proteins affect HIF-1 transactivation. Other HCMV-encoded receptors GPCRs also reported to be expressed on the virion, like UL33 [58], may play a role in modulating this pathway as well.

Previously, we have shown that VEGF promoter activation by US28 was mediated through activation of G-proteins, more specifically Gα_q_ and Gβγ, followed by activation of protein kinases p38 and p44/42 MAPK [18]. Subsequently, we confirmed the involvement of STAT3 in US28-mediated VEGF promoter activity and showed that COX-2 inhibitor celecoxib is able to partially decrease US28-induced VEGF secretion [20, 21]. Here, we show that US28-induced VEGF promoter activity and secretion is mediated through HIF-1 transactivation via activation of Gα_q_, CaMKII and Akt/mTOR (Figure 3). Pharmacological inhibition of the previously described NF-κB and STAT3/IL-6 pathway signaling did not significantly inhibit US28-dependent HIF-1 activation, indicating this is an independent and previously unrecognized signaling cascade. As the VEGF promoter region contains both HRE and STAT3 response elements, US28 may facilitate VEGF expression and thus angiogenesis, through at least two distinct mechanisms [59]. These findings accentuate the complexity and promiscuity of the signaling mechanisms of this viral chemokine receptor. The involvement of Akt/mTOR cascade in canonical HIF-1 regulation is well established. This signaling cascade has been reported to promote HIF-1α activity by stimulating mRNA translation, thus elevating protein levels, whilst HIF-1α protein stability is unchanged [60, 61]. Our data suggests that US28 utilizes a similar mechanism for HIF-1α regulation by increasing the rate of mRNA translation to protein through Akt/mTOR-dependent pathways. *HIF1A* mRNA levels are unchanged in US28-expressing cells, while the protein levels and transcriptional activity are increased (Figure 2) and can be attenuated by the mTOR inhibitor rapamycin and Akt inhibitor GSK690693 (Figure 3). In US28-expressing glioblastoma cells we detected elevated levels of active (phosphorylated at Ser-473) Akt (Figure 4), further implicating the involvement of the Akt/mTOR pathway in US28 oncomodulatory signaling. Akt/mTOR signaling is frequently regulated by PI3K activation, which can be induced by receptor tyrosine kinase growth factor or GPCR signaling. Interestingly, the US28 mediated effects appear to be PI3K independent (Figure 3 and 4). As CaMKII inhibition results in attenuated HIF-1 transcriptional activity, Akt phosphorylation and cell proliferation (Figure 3, 4 and 5 respectively), we suggest that US28 may activate CaMKII which subsequently orchestrates activation of the Akt/mTOR cascade to activate HIF-1α. Our data confirms previous research, which demonstrated that CaMKII stimulates Akt phosphorylation, HIF-1 protein stability and VEGF secretion [62-64].

PKM2 plays an enigmatic dual role in cellular metabolism and proliferation. In healthy cells primarily PKM2 exists in a tetrameric state, functioning as an essential enzyme in glucose metabolism [65]. Intriguingly, its enzymatic role can be changed by oncogenic signaling that causes phosphorylation and subsequent destabilization of the tetramer, thus promoting less enzymatically active PKM2 dimers [66]. As a consequence of reduced PKM2 enzyme activity, accumulation of PEP, elevated levels of aerobic glycolysis and lactate production occurs [26, 38]. Only in its dimeric form PKM2 can translocate into the nucleus and act as a transcriptional co-activator, enhancing activity of STAT3 [28], β-catenin [67] and HIF-1 [68]. This study revealed that US28 expression in NIH-3T3 cells strongly stimulates the Tyr105 phosphorylation and related dimeric state of PKM2. Similar trends, although to a lesser extent are observed after expression of US28 in U251 cells (Figure 4). It is noteworthy that basal PKM2 protein levels are considerably higher (over 10-fold, data not shown) in U251 glioma cells compared to the NIH-3T3 cells, which might indicate that U251 cells rely on aerobic glycolysis for glucose metabolism, and have previously undergone metabolic reprogramming. Nevertheless, US28 expression did have an effect on PKM2 phosphorylation and dimerization in the glioblastoma cells, suggesting that US28 affects glucose metabolism of HCMV-infected tumor cells. The expression and function of PKM2 are closely intertwined with HIF-1 in feedforward loops, whereas *HIF1A* is a target gene of the PKM2/STAT3 pathway, *PKM2* expression is on its turn regulated by the HIF-1 transcription complex [27]. The finding that total protein expression levels of PKM2 are increased in US28-expressing cells is in line with the reported positive feedback loop between HIF-1 and PKM2, since an increase in HIF-1 activity appears to be related to an increase in PKM2 expression. Very recently it was reported that ORF74 (i.e KSHV-encoded vGPCR) signaling stimulates HIF-1α and PKM2 expression levels [69]. Since we now observe similar effects in HCMV-infected and US28-expressing cells, this suggests a conserved herpesvirus mechanism to exploit the host's cell machinery by means of viral GPCR signaling.

Although activation of proliferative and angiogenic signaling by US28 is well established, the effects of this viral GPCR on cell metabolism were not assessed in previous studies. Here, we characterized the potential of US28 to regulate transcription of genes involved in glucose metabolism and evaluated the effects on aerobic glycolysis. We found that US28 expression in NIH-3T3 cells, increases mRNA levels of the glucose transporter *GLUT1* and metabolic enzyme *GAPDH*. Furthermore, US28 expression in U251 cells enhances lactate secretion, which may be indicative for increased glycolysis and a tumor-promoting metabolic state. Overall, these findings support the hypothesis that US28 signaling has the potential to drive metabolic reprogramming of (pre-) malignant cells in order to support an onco-proliferative phenotype.

**Figure 7:**
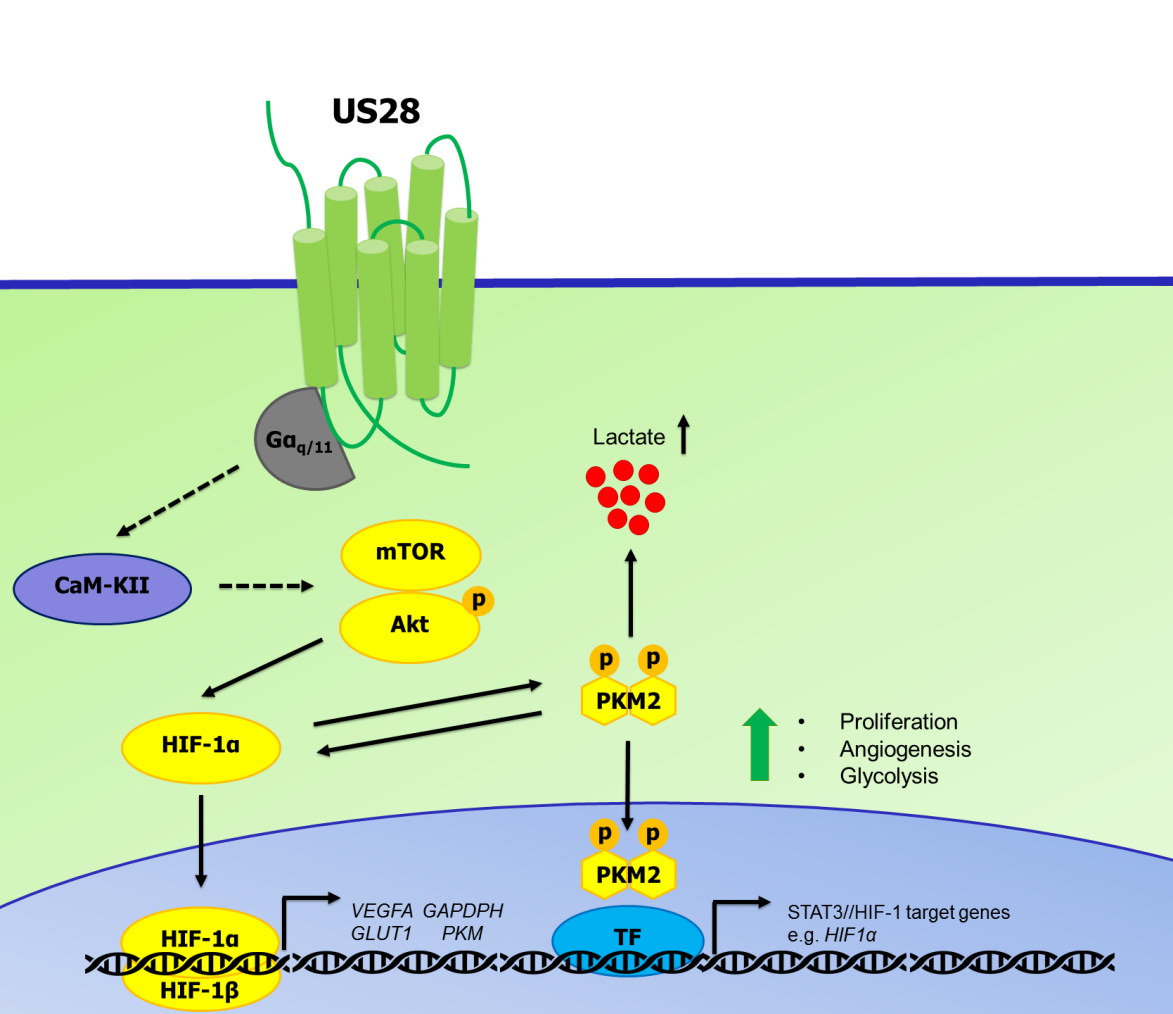
Proposed mechanism of US28 induced activation of the HIF-1α/PKM2 feedforward loop US28 constitutively stimulates Gα_q_-dependent signaling cascades resulting in CaMKII activity and subsequent stimulation of the Akt/mTOR cascade to increase HIF-1α translation and/or stability. HIF-1α translocates to the nucleus and dimerizes with HIF-1β to form a functional HIF-1 transcription factor that binds HRE regions to regulate HIF-1 target genes supporting proliferation, angiogenesis and metabolic reprogramming. US28 signaling modulates PKM2 phosphorylation and rearrangement into enzymatically less active, but transcriptional active dimers. Dimeric PKM2 is linked with glycolysis and thus increased levels lactate production and secretion. PKM2 dimers and HIF-1α engage in a feedforward loop supporting a proliferative, angiogenic and glycolytic phenotype.

Our previous work has demonstrated the importance of the constitutive activity of US28 in enhancing proliferative and angiogenic signaling and contributing to HCMV-associated malignancies [18, 20, 21, 70, 71]. The current data indicate that US28 is also able to impinge into the signaling module regulating HIF-1 activity. Here, we propose another novel mechanism by which US28 is able to promote tumorigenesis by feeding into the HIF-1/PKM2 axis (Figure 7). US28 constitutively signals through Gα_q_ which results in activation of CaMKII, stimulating Akt activity by Ser-473 phosphorylation and subsequent mTOR-dependent HIF-1α upregulation. This mechanism is responsible for increased translocation of HIF-1α to the nucleus, dimerizing with HIF-1β and binding to HRE regions of angiogenic and glycolytic target genes like *VEGF-A*, *GLUT1* and *GAPDH*. Increased secretion of VEGF-A, as was reported before, leads to angiogenesis *in vitro* and *in vivo* [18]. Furthermore, active HIF-1α engages with phosphorylated (Tyr105) and dimeric PKM2 in a feedforward loop to sustain cell proliferation, angiogenesis and glycolysis. Phosphorylation at Tyr-105 of PKM2 is mediated by US28, leads to less energy efficient conversion of pyruvate on one hand and translocation of PKM2 to the nucleus to function as a transcriptional co-activator of transcription factors like HIF-1 and STAT3.

In conclusion, in this study we show that the HCMV-encoded GPCR US28 stimulates the HIF-1/PKM2 feedforward loop through concerted activation of Gα_q_, CaMKII and the Akt/mTOR cascade, regulating proliferation, angiogenesis and reprogramming of glucose metabolism. These findings further substantiate a role for this viral GPCR in HCMV-associated malignancies.

## MATERIALS AND METHODS

### Cell culture

All experiments were performed under normoxic conditions. NIH-3T3 cells stably transfected with mock vector (empty pcDEF3 expression vector), US28-WT or US28-R^129^A were cultured as previously described [18]. The human embryonic kidney cell line HEK293T and the human malignant glioblastoma cell line U251 were cultured in DMEM (Sigma-Aldrich) supplemented with 10% fetal bovine serum, heat-inactivated bovine serum (Gibco), respectively. Human foreskin fibroblasts (HFF) were cultured in MEM Eagle with Earle's salts and sodium bicarbonate (Sigma-Aldrich) supplemented with 0.1 mmol/L non-essential amino acids (PAA), 2 mmol/L _L_-glutamine and 10% fetal bovine serum. 50 IU/ml penicillin and 50 μg/ml streptomycin (PAA) was added to all culture media and cells were grown at 37°C in a humidified atmosphere with 5% CO_2_. Transient transfections were performed using the polyethylenimine (PEI) method [72]. A U251 cell line with inducible US28 expression (U251-iUS28) was generated by lentiviral transduction. US28 (VHL/E strain) was subcloned in the pLenti6.3/To/V5-DEST vector by recombination cloning (Thermo Scientific). For lentivirus production, US28- pLenti6.3/To/V5-DEST or pLenti3.3/TR (Thermo Scientific) was co-transfected with pRSV-REV, pMDLg/pRRE and pMD2.g packaging vectors in HEK293T cells. 24 hours post-transfection the transfection reagents are removed from the medium and fresh culture medium is added. After 24/48 hours of incubation, medium containing lentiviral particles was collected from the transfected HEK293T cells. The lentivirus medium centrifuged to remove cell debris, sterile-filtered and stored at 4°C. U251 cells were transduced with Tet-repressor encoding lentiviral particles and incubated O/N. The lentivirus containing medium was removed and cells were incubated for 24 hours in regular culture medium. This was followed by a second transduction with lentiviral particles encoding US28 with inducible promoter, which were incubated for 24 hours. US28 expression was induced by addition of 1 μg/mL doxycycline (Sigma-Aldrich) to the culture medium. The clinical HCMV strain Titan WT and the deletion mutant Titan ΔUS28, previously described in [18] were used to infect U251 and HFF cells with a MOI of 2. In order to synchronize the infection cycle, the virus was removed 2 hours post infection.

### Materials

Recombinant human CX3CL1 was obtained from Peprotech. Recombinant human IL-6 protein was obtained from R&D systems. Acriflavine (HIF-1 inhibitor), KN-93 (CaMKII inhibitor), GSK690693 (pan-Akt inhibitor), BAY 11-7082 (NF-κB signaling inhibitor), U73122 hydrate (pan-PLC inhibitor), Stattic (STAT3 inhibitor), LY-294002 hydrochloride (PI3K inhibitor), Sodium oxamate (LDHA inhibitor), pertussis toxin (PTx,Gα_i/0_ inhibitor), H-89 (PKA/ROCK inhibitor), Y-27632 (ROCK inhibitor) and CoCl_2_ were all obtained from Sigma-Aldrich. Rapamycin (mTORC1 inhibitor) was purchased from Cayman Chemical. 17-AAG (HSP90 inhibitor) and P6 (pan-JAK inhibitor), Bisindolylmaleimide I (PKC inhibitor) were purchased from Calbiochem. The Gα_q_-inhibitor UBO-QIC was purchased from the Institute of Pharmaceutical Biology, University of Bonn. Gallein (βγ-subunit inhibitor) was obtained from Tocris.

### Radioligand binding

Expression of US28 in stably transfected NIH-3T3 and U251-iUS28 cells was confirmed using ^125^I-CCL5 binding (specific binding was determined using CX3CL1 10^−7^ M) as was described previously [19]. When required, 1 μg/mL doxycycline was added to the medium for at least 24 hours to induce US28 expression in U251-iUS28 cells.

### VEGF ELISA

NIH-3T3-Mock, NIH-3T3-US28 (1.10^5^) or U251-iUS28 (1.5.10^5^) cells were seeded in regular growth medium in a 6-well plate. The next day cells were synchronized by serum starvation (0.5% bovines serum for NIH-3T3 cells, 0% serum for U251). After O/N synchronization, the medium was refreshed with serum starvation medium containing inhibitor or vehicle control. After 24 or 48 hour incubation the conditioned medium was collected, centrifuged and the supernatant was used in a mouse-VEGF or human-VEGF Quantikine kit (R&D Systems), according to manufacturer's procedures.

### Reporter gene analysis

HEK293T cells were transfected with US28 and either VEGF promoter luciferase reporter gene as described earlier [18] or HIF-1 reporter gene. For the latter, 1.10^6^ cells were transfected with US28-pcDEF3 (25-100 ng), 1 μg pTL-HIF-1-luc vector (Affymetrix) supplemented with empty pcDEF3 vector to a total of 2 μg. Transfected cells were seeded in white bottom 96-well assay plates. Immediately after transfection, cells were treated with Cobalt Chloride (150 μM), Acriflavine (1 μM), 17-AAG (1 μM), Rapamycin (1 μM), UBO-QIC (1 μM), pertussis toxin (100 ng/mL), KN-93 (10 μM), hIL-6 (6 pg/mL), GSK690693 (50 nM), Bisindolylmaleimide I (5 μM), H-89 (10 μM), Y27632 (10 μM), BpV (1 μM), P6 (1 μM), gallein (10 μM), U73122 (5 μM), LY-294002 (10 μM), Stattic (10 μM) or 2-APB (100 μM) overnight. Luciferase activities were measured 24 hours after transfection. For infection experiments, 2.10^5^ HFF and U251 cells were seeded and grown overnight in a 6-well plate. The cells were transfected with 900 ng pTL-HIF-1-luc vector (Affymetrix) and 100 ng constitutive renilla luciferase-pcDNA3.1 vector. The constitutive renilla luciferase activity was used to correct for changes in basal transcriptional activity after infection. 6 hours post transfection the cells were mock, HCMV Titan WT or HCMV Titan ΔUS28 infected with an MOI of 2 and grown overnight. The cells were reseeded in a white bottom 96-well assay plate and luciferase activity was measured 48 hours post-infection.

### Quantitative real-time-PCR

NIH-3T3 cells were cultured in a 6-well plate and harvested after 24 hours. The cells were centrifuged and the pellet was used to isolate RNA with RNeasy kit (Qiagen). Subsequently cDNA was prepared by the use of iScript reverse transcriptase kit (Bio-Rad) according to manufacturer's instructions. The applied qPCR primers are: *HIF1A* F:TCATCAGTTGCCACTTCCCCAC, R:CCGTCATCTGTTAGCACCATCA; *GAPDH* F:TCAACGGCACAGTCAAGG, R:ACTCCACGACATACTCAGC; *GLUT1* F:GAGACCAAAGCGTGGTGAGT, R:GCAGTTCGGCTATAACACTGG; *VEGFA* F:GGCTCTTCTCGCTCCGTAGTAG, R:CCTCTCCTCTTCCTTCTCTTCCTC; *ACTB* F:GGCTGTATTCCCCTCCATCG, R:CCAGTTGGTAACAATGCCATGT. PCR reactions were performed using SYBR Green mix with MyiQ Real-Time PCR detection system (Bio-Rad). The obtained Ct values of the genes of interest were normalized to the internal control *ACTB* and relative expression levels were determined using the 2^−ΔΔCt^ method.

### Western blot

NIH-3T3 cells (1.10^5^) cells were grown on a 6-well plate on full medium. After overnight incubation the cells were either lysed (for detection of HIF-1α) or synchronized by replacing the medium with 0.5% (v/v) bovine serum DMEM and incubated for 24 hours before lysis. U251 cells (1.5.10^5^) with inducible US28 expression (U251-iUS28) were seeded on a 6-well plate on full medium. Cells were synchronized by serum starvation with 0% FBS DMEM and grown overnight, medium was replaced with 0% FBS DMEM with or without 1 μg/mL doxycycline for US28 expression, and if necessary, signaling pathway inhibitors, UBO-QIC (1 μM), KN-93 (10 μM), GSK690693 (50 nM), LY-294002 (10 μM), BAY 11-7082 (10 μM). After synchronization (NIH-3T3) or 48 hour induction of US28 expression and treatment (U251-iUS28) cells were lysed with RIPA buffer or native lysis buffer (25 mM Tris-HCl (pH 7.4), 150 mM NaCl, 1 mM EDTA, 1% (v/v) NP40 and 5% (v/v) glycerol) supplemented with 1 mM NaF, 1 mM NaVO_4_ and cOmplete protease inhibitor cocktail (Roche), sonicated and centrifuged to remove insoluble cell debris. Protein content of the lysates was determined using a BCA protein estimation assay according to manufacturer's instructions (Thermo Scientific). For cross-linking experiments, cells were lysed using native lysis buffer, cross-linking was carried out on native lysates through incubation with 0.25% formaldehyde (Sigma) solution in PBS for 10 minutes at 25°C. Cross-linking reaction was quenched and sample was denatured and reduced by addition of 6X laemmli sample and 5 minute incubation at 65°C. Western blot was performed according to standard procedure and blotted against HIF-1α (Millipore, #MAB5382 for NIH-3T3 and BD Transduction, #610959 for U251), pPKM2-Y105 #3827, PKM2 total #4053, pAkt-S473 #4060, Akt #9272 (all Cell Signaling Technology) and β-actin #A5316 (Sigma). The Western blot images were acquired by the use of Chemi Doc imager (Bio-Rad) and quantified by densitometry using Image lab Software (Bio-Rad).

### Thymidine incorporation assay

Cell proliferation in NIH-3T3 cells was measured using ^3^H-thymidine incorporation. Cells were grown in full medium and next day put on medium containing 0.5% bovine serum overnight with or without 1 μM Acriflavine, before labeling in DMEM supplemented with 0.5% bovine serum containing 1 μCi/ml ^3^H-thymidine (Perkin-Elmer). The next day cells were fixed with methanol, washed, lysed and radioactivity was measured with a scintillation counter.

### Lactate secretion and protein estimation

U251-iUS28 cells (1.5.10^5^) were seeded in 6-well plates and grown O/N in full growth culture medium. The cells were synchronized by serum starvation (0% FBS/DMEM) for at least 16 hours. The medium was aspirated and 0% FBS/DMEM with/without doxycycline (1 μg/mL) to induce US28 expression and/or inhibitor (UBO-QIC (1 μM), KN-93 (10 μM), or LY-294002 (10 μM)) was added to the cells. Medium was collected and centrifuged after 48 hours of conditioning, the supernatant was stored at −20^°^C until the L-Lactate assay was performed. The cells of each condition were lysed and the protein content was determined using a BCA protein estimation assay kit (Pierce). To determine the L-lactate levels in the conditioned media a glycolysis cell-based assay kit (Cayman Chemical) was used according to the manufacturer's instructions. Absorbance was measured at 490 nm with the PowerWave spectrophotometer (BioTek). Absolute L-lactate levels in the medium were determined using a L-lactate standard curve. The results were normalized to the protein content of each sample.

### Immunofluorescence

HCMV Titan-infected cells were fixed 48 hours post-infection using 4% formaldehyde (Sigma). Fixed cells were blocked using 5% (w/v) BSA/PBS (PAA) and immunofluorescence was performed with mouse anti-cytomegalovirus antibody (MAB810R, Millipore), rabbit anti-US28 antibody kindly provided by Alberto Fraille Ramos [21], and the secondary anti-mouse Alexa Fluor^®^ 488-conjugated and anti-rabbit Alexa Fluor^®^ 568-conjugated antibodies. Imaging was carried out using a Olympus FSX-100 microscope.

### Data analyses

All data was obtained from at least three independent experiments in triplicates. All bar graphs and statistical analyses were obtained with GraphPad Prism 6 software (GraphPad software Inc, San Diego) ANOVA analyses with multiple comparisons test and Tukey correction were performed or multiple t tests with Holm-Sidak correction to statistically (α=0.05) compare different groups. Bars and errors in the graphs as well as data in the text represent the mean ± SEM.

## References

[1] Gandhi MK, Khanna R (2004). Human cytomegalovirus: clinical aspects, immune regulation, and emerging treatments. The Lancet Infectious diseases.

[2] White MK, Gorrill TS, Khalili K (2006). Reciprocal transactivation between HIV-1 and other human viruses. Virology.

[3] Fischer SA (2008). Emerging viruses in transplantation: there is more to infection after transplant than CMV and EBV. Transplantation.

[4] Nakase H, Honzawa Y, Toyonaga T, Yamada S, Minami N, Yoshino T, Matsuura M (2014). Diagnosis and treatment of ulcerative colitis with cytomegalovirus infection: importance of controlling mucosal inflammation to prevent cytomegalovirus reactivation. Intestinal research.

[5] Vischer HF, Siderius M, Leurs R, Smit MJ (2014). Herpesvirus-encoded GPCRs: neglected players in inflammatory and proliferative diseases?. Nat Rev Drug Discov.

[6] Harkins L, Volk AL, Samanta M, Mikolaenko I, Britt WJ, Bland KI, Cobbs CS (2002). Specific localisation of human cytomegalovirus nucleic acids and proteins in human colorectal cancer. Lancet.

[7] Chen HP, Jiang JK, Chen CY, Chou TY, Chen YC, Chang YT, Lin SF, Chan CH, Yang CY, Lin CH, Lin JK, Cho WL, Chan YJ (2012). Human cytomegalovirus preferentially infects the neoplastic epithelium of colorectal cancer: a quantitative and histological analysis. J Clin Virol.

[8] Harkins LE, Matlaf LA, Soroceanu L, Klemm K, Britt WJ, Wang W, Bland KI, Cobbs CS (2010). Detection of human cytomegalovirus in normal and neoplastic breast epithelium. Herpesviridae.

[9] Samanta M, Harkins L, Klemm K, Britt WJ, Cobbs CS (2003). High prevalence of human cytomegalovirus in prostatic intraepithelial neoplasia and prostatic carcinoma. J Urol.

[10] Giuliani L, Jaxmar T, Casadio C, Gariglio M, Manna A, D'Antonio D, Syrjanen K, Favalli C, Ciotti M (2007). Detection of oncogenic viruses SV40, BKV, JCV, HCMV, HPV and p53 codon 72 polymorphism in lung carcinoma. Lung Cancer.

[11] Rahbar A, Orrego A, Peredo I, Dzabic M, Wolmer-Solberg N, Straat K, Stragliotto G, Soderberg-Naucler C (2013). Human cytomegalovirus infection levels in glioblastoma multiforme are of prognostic value for survival. J Clin Virol.

[12] Fornara O, Bartek J, Rahbar A, Odeberg J, Khan Z, Peredo I, Hamerlik P, Bartek J, Stragliotto G, Landazuri N, Soderberg-Naucler C (2016). Cytomegalovirus infection induces a stem cell phenotype in human primary glioblastoma cells: prognostic significance and biological impact. Cell Death Differ.

[13] Cobbs CS (2011). Evolving evidence implicates cytomegalovirus as a promoter of malignant glioma pathogenesis. Herpesviridae.

[14] Soroceanu L, Matlaf L, Bezrookove V, Harkins L, Martinez R, Greene M, Soteropoulos P, Cobbs CS (2011). Human cytomegalovirus US28 found in glioblastoma promotes an invasive and angiogenic phenotype. Cancer research.

[15] Matlaf LA, Harkins LE, Bezrookove V, Cobbs CS, Soroceanu L (2013). Cytomegalovirus pp71 protein is expressed in human glioblastoma and promotes pro-angiogenic signaling by activation of stem cell factor. PloS one.

[16] Pandey JP, Kaur N, Costa S, Amorim J, Nabico R, Linhares P, Vaz R, Viana-Pereira M, Reis RM (2014). Immunoglobulin genes implicated in glioma risk. Oncoimmunology.

[17] Slinger E, Langemeijer E, Siderius M, Vischer HF, Smit MJ (2011). Herpesvirus-encoded GPCRs rewire cellular signaling. Mol Cell Endocrinol.

[18] Maussang D, Verzijl D, van Walsum M, Leurs R, Holl J, Pleskoff O, Michel D, van Dongen GA, Smit MJ (2006). Human cytomegalovirus-encoded chemokine receptor US28 promotes tumorigenesis.

[19] Casarosa P, Bakker RA, Verzijl D, Navis M, Timmerman H, Leurs R, Smit MJ (2001). Constitutive signaling of the human cytomegalovirus-encoded chemokine receptor US28. The Journal of biological chemistry.

[20] Maussang D, Langemeijer E, Fitzsimons CP, Stigter-van Walsum M, Dijkman R, Borg MK, Slinger E, Schreiber A, Michel D, Tensen CP, van Dongen GA, Leurs R, Smit MJ (2009). The human cytomegalovirus-encoded chemokine receptor US28 promotes angiogenesis and tumor formation via cyclooxygenase-2. Cancer research.

[21] Slinger E, Maussang D, Schreiber A, Siderius M, Rahbar A, Fraile-Ramos A, Lira SA, Soderberg-Naucler C, Smit MJ (2010). HCMV-encoded chemokine receptor US28 mediates proliferative signaling through the IL-6-STAT3 axis. Science signaling.

[22] Baryawno N, Rahbar A, Wolmer-Solberg N, Taher C, Odeberg J, Darabi A, Khan Z, Sveinbjornsson B, FuskevAg OM, Segerstrom L, Nordenskjold M, Siesjo P, Kogner P, Johnsen JI, Soderberg-Naucler C (2011). Detection of human cytomegalovirus in medulloblastomas reveals a potential therapeutic target. The Journal of clinical investigation.

[23] Eswarappa SM, Fox PL (2015). Antiangiogenic VEGF-Ax: A New Participant in Tumor Angiogenesis. Cancer research.

[24] Semenza GL (2012). Hypoxia-inducible factors: mediators of cancer progression and targets for cancer therapy. Trends in pharmacological sciences.

[25] Hanahan D, Weinberg RA (2011). Hallmarks of cancer: the next generation. Cell.

[26] Wong N, De Melo J, Tang D (2013). PKM2, a Central Point of Regulation in Cancer Metabolism. Int J Cell Biol.

[27] Demaria M, Poli V (2012). PKM2, STAT3 and HIF-1alpha: The Warburg's vicious circle. JAKSTAT.

[28] Gao X, Wang H, Yang JJ, Liu X, Liu ZR (2012). Pyruvate kinase M2 regulates gene transcription by acting as a protein kinase. Mol Cell.

[29] Filipp FV (2013). Cancer metabolism meets systems biology: Pyruvate kinase isoform PKM2 is a metabolic master regulator. J Carcinog.

[30] Lee K, Zhang H, Qian DZ, Rey S, Liu JO, Semenza GL (2009). Acriflavine inhibits HIF-1 dimerization, tumor growth, and vascularization.

[31] Ke Q, Costa M (2006). Hypoxia-inducible factor-1 (HIF-1). Mol Pharmacol.

[32] Yuan Y, Hilliard G, Ferguson T, Millhorn DE (2003). Cobalt inhibits the interaction between hypoxia-inducible factor-alpha and von Hippel-Lindau protein by direct binding to hypoxia-inducible factor-alpha. The Journal of biological chemistry.

[33] Hamanaka RB, Chandel NS (2012). Targeting glucose metabolism for cancer therapy. The Journal of experimental medicine.

[34] Adekola K, Rosen ST, Shanmugam M (2012). Glucose transporters in cancer metabolism. Current opinion in oncology.

[35] Cerella C, Dicato M, Diederich M (2014). Modulatory roles of glycolytic enzymes in cell death. Biochem Pharmacol.

[36] Wenger RH, Stiehl DP, Camenisch G (2005). Integration of oxygen signaling at the consensus HRE. Sci STKE.

[37] Pore N, Jiang Z, Shu HK, Bernhard E, Kao GD, Maity A (2006). Akt1 activation can augment hypoxia-inducible factor-1alpha expression by increasing protein translation through a mammalian target of rapamycin-independent pathway. Mol Cancer Res.

[38] Wong N, Ojo D, Yan J, Tang D (2015). PKM2 contributes to cancer metabolism. Cancer letters.

[39] Hitosugi T, Kang S, Vander Heiden MG, Chung TW, Elf S, Lythgoe K, Dong S, Lonial S, Wang X, Chen GZ, Xie J, Gu TL, Polakiewicz RD, Roesel JL, Boggon TJ, Khuri FR (2009). Tyrosine phosphorylation inhibits PKM2 to promote the Warburg effect and tumor growth. Science signaling.

[40] Gao X, Wang H, Yang JJ, Chen J, Jie J, Li L, Zhang Y, Liu ZR (2013). Reciprocal regulation of protein kinase and pyruvate kinase activities of pyruvate kinase M2 by growth signals. The Journal of biological chemistry.

[41] Warburg O (1956). On the origin of cancer cells. Science.

[42] Sanzey M, Abdul Rahim SA, Oudin A, Dirkse A, Kaoma T, Vallar L, Herold-Mende C, Bjerkvig R, Golebiewska A, Niclou SP (2015). Comprehensive analysis of glycolytic enzymes as therapeutic targets in the treatment of glioblastoma. PloS one.

[43] McFarlane S, Nicholl MJ, Sutherland JS, Preston CM (2011). Interaction of the human cytomegalovirus particle with the host cell induces hypoxia-inducible factor 1 alpha. Virology.

[44] Fraile-Ramos A, Kledal TN, Pelchen-Matthews A, Bowers K, Schwartz TW, Marsh M (2001). The human cytomegalovirus US28 protein is located in endocytic vesicles and undergoes constitutive endocytosis and recycling. Molecular biology of the cell.

[45] Semenza GL (2007). Life with oxygen. Science.

[46] Semenza GL (2010). Defining the role of hypoxia-inducible factor 1 in cancer biology and therapeutics. Oncogene.

[47] Cai Q, Murakami M, Si H, Robertson ES (2007). A potential alpha-helix motif in the amino terminus of LANA encoded by Kaposi's sarcoma-associated herpesvirus is critical for nuclear accumulation of HIF-1alpha in normoxia. J Virol.

[48] Cai QL, Knight JS, Verma SC, Zald P, Robertson ES (2006). EC5S ubiquitin complex is recruited by KSHV latent antigen LANA for degradation of the VHL and p53 tumor suppressors. PLoS pathogens.

[49] Sodhi A, Montaner S, Patel V, Zohar M, Bais C, Mesri EA, Gutkind JS (2000). The Kaposi's sarcoma-associated herpes virus G protein-coupled receptor up-regulates vascular endothelial growth factor expression and secretion through mitogen-activated protein kinase and p38 pathways acting on hypoxia-inducible factor 1alpha. Cancer research.

[50] Shin YC, Joo CH, Gack MU, Lee HR, Jung JU (2008). Kaposi's sarcoma-associated herpesvirus viral IFN regulatory factor 3 stabilizes hypoxia-inducible factor-1 alpha to induce vascular endothelial growth factor expression. Cancer Res.

[51] Yoo YG, Oh SH, Park ES, Cho H, Lee N, Park H, Kim DK, Yu DY, Seong JK, Lee MO (2003). Hepatitis B virus X protein enhances transcriptional activity of hypoxia-inducible factor-1alpha through activation of mitogen-activated protein kinase pathway. J Biol Chem.

[52] Nasimuzzaman M, Waris G, Mikolon D, Stupack DG, Siddiqui A (2007). Hepatitis C virus stabilizes hypoxia-inducible factor 1alpha and stimulates the synthesis of vascular endothelial growth factor. J Virol.

[53] Darekar S, Georgiou K, Yurchenko M, Yenamandra SP, Chachami G, Simos G, Klein G, Kashuba E (2012). Epstein-Barr virus immortalization of human B-cells leads to stabilization of hypoxia-induced factor 1 alpha, congruent with the Warburg effect. PLoS One.

[54] Kondo S, Seo SY, Yoshizaki T, Wakisaka N, Furukawa M, Joab I, Jang KL, Pagano JS (2006). EBV latent membrane protein 1 up-regulates hypoxia-inducible factor 1alpha through Siah1-mediated down-regulation of prolyl hydroxylases 1 and 3 in nasopharyngeal epithelial cells. Cancer Res.

[55] Nakamura M, Bodily JM, Beglin M, Kyo S, Inoue M, Laimins LA (2009). Hypoxia-specific stabilization of HIF-1alpha by human papillomaviruses. Virology.

[56] Rahbar A, Cederarv M, Wolmer-Solberg N, Tammik C, Stragliotto G, Peredo I, Fornara O, Xu X, Dzabic M, Taher C, Skarman P, Soderberg-Naucler C (2016). Enhanced neutrophil activity is associated with shorter time to tumor progression in glioblastoma patients. Oncoimmunology.

[57] Penfold ME, Schmidt TL, Dairaghi DJ, Barry PA, Schall TJ (2003). Characterization of the rhesus cytomegalovirus US28 locus. Journal of virology.

[58] Fraile-Ramos A, Pelchen-Matthews A, Kledal TN, Browne H, Schwartz TW, Marsh M (2002). Localization of HCMV UL33 and US27 in endocytic compartments and viral membranes. Traffic.

[59] Pages G, Pouyssegur J (2005). Transcriptional regulation of the Vascular Endothelial Growth Factor gene—a concert of activating factors. Cardiovasc Res.

[60] Laughner E, Taghavi P, Chiles K, Mahon PC, Semenza GL (2001). HER2 (neu) signaling increases the rate of hypoxia-inducible factor 1alpha (HIF-1alpha) synthesis: novel mechanism for HIF-1-mediated vascular endothelial growth factor expression. Mol Cell Biol.

[61] Semenza GL (2010). HIF-1: upstream and downstream of cancer metabolism. Current opinion in genetics & development.

[62] Lee KH (2010). CaMKII Inhibitor KN-62 Blunts Tumor Response to Hypoxia by Inhibiting HIF-1alpha in Hepatoma Cells. Korean J Physiol Pharmacol.

[63] Daft PG, Yang Y, Napierala D, Zayzafoon M (2015). The growth and aggressive behavior of human osteosarcoma is regulated by a CaMKII-controlled autocrine VEGF signaling mechanism. PloS one.

[64] Rokhlin OW, Taghiyev AF, Bayer KU, Bumcrot D, Koteliansk VE, Glover RA, Cohen MB (2007). Calcium/calmodulin-dependent kinase II plays an important role in prostate cancer cell survival. Cancer Biol Ther.

[65] Ashizawa K, Willingham MC, Liang CM, Cheng SY (1991). *In vivo* regulation of monomer-tetramer conversion of pyruvate kinase subtype M2 by glucose is mediated via fructose 1,6-bisphosphate. The Journal of biological chemistry.

[66] Mazurek S, Eigenbrodt E (2003). The tumor metabolome. Anticancer research.

[67] Yang W, Xia Y, Ji H, Zheng Y, Liang J, Huang W, Gao X, Aldape K, Lu Z (2011). Nuclear PKM2 regulates beta-catenin transactivation upon EGFR activation. Nature.

[68] Luo W, Hu H, Chang R, Zhong J, Knabel M, O'Meally R, Cole RN, Pandey A, Semenza GL (2011). Pyruvate kinase M2 is a PHD3-stimulated coactivator for hypoxia-inducible factor 1. Cell.

[69] Ma T, Patel H, Babapoor-Farrokhran S, Franklin R, Semenza GL, Sodhi A, Montaner S (2015). KSHV induces aerobic glycolysis and angiogenesis through HIF-1-dependent upregulation of pyruvate kinase 2 in Kaposi's sarcoma. Angiogenesis.

[70] Langemeijer EV, Slinger E, de Munnik S, Schreiber A, Maussang D, Vischer H, Verkaar F, Leurs R, Siderius M, Smit MJ (2012). Constitutive beta-catenin signaling by the viral chemokine receptor US28. PloS one.

[71] Bongers G, Maussang D, Muniz LR, Noriega VM, Fraile-Ramos A, Barker N, Marchesi F, Thirunarayanan N, Vischer HF, Qin L, Mayer L, Harpaz N, Leurs R, Furtado GC, Clevers H, Tortorella D (2010). The cytomegalovirus-encoded chemokine receptor US28 promotes intestinal neoplasia in transgenic mice. The Journal of clinical investigation.

[72] Schlaeger EJ, Christensen K (1999). Transient gene expression in mammalian cells grown in serum-free suspension culture. Cytotechnology.

